# Pre-operative echocardiography among patients with coronary artery disease in the United States Veterans Affairs healthcare system: A retrospective cohort study

**DOI:** 10.1186/s12872-016-0357-5

**Published:** 2016-09-05

**Authors:** Emily B. Levitan, Laura A. Graham, Javier A. Valle, Joshua S. Richman, Robert Hollis, Carla N. Holcomb, Thomas M. Maddox, Mary T. Hawn

**Affiliations:** 1 Department of Epidemiology, University of Alabama at Birmingham, 35294-0022 Birmingham, AL USA; 2 Birmingham Veterans Affairs Medical Center, Birmingham, AL USA; 3 Division of Cardiology, University of Colorado, Denver, CO USA; 4 Department of Surgery, University of Alabama at Birmingham, Birmingham, AL USA; 5 Veterans Affairs Eastern Colorado Health Care System, Denver, CO USA; 6 Department of Surgery, Stanford University, Stanford, CA USA

**Keywords:** Echocardiography, Surgery, Major adverse cardiac events

## Abstract

**Background:**

Echocardiography is not recommended for routine pre-surgical evaluation but may have value for patients at high risk of major adverse cardiovascular events (MACE). The objective of this study was to evaluate whether pre-operative echocardiography is associated with lower risk of post-operative MACE among patients with coronary artery disease.

**Methods:**

Using administrative and registry data, we examined associations of echocardiography within 3 months prior to surgery with postoperative MACE (myocardial infarction, revascularization, or death within 30 days) among patients with coronary artery disease undergoing elective, non-cardiac surgeries in the United States Veterans Affairs healthcare system in 2000–2012.

**Results:**

Echocardiography preceded 4,378 (16.4 %) of 26,641 surgeries. MACE occurred within 30 days following 944 (3.5 %) surgeries. A 10 % higher case-mix adjusted rate of pre-operative echocardiography assessed at the hospital level was associated with a hospital-level risk of MACE that was 1.0 % (95 % confidence interval [CI] 0.1 %, 2.0 %) higher overall and 1.7 % (95 % CI 0.2 %, 3.2 %) higher among patients with recent myocardial infarction, valvular heart disease, or heart failure. At the patient level, pre-operative echocardiography was associated with an odds ratio for MACE of 1.9 (95 % CI 1.7, 2.2) overall and 1.8 (95 % CI 1.5, 2.2) among patients with recent myocardial infarction, valvular heart disease, or heart failure adjusting for MACE risk factors.

**Conclusions:**

Pre-operative echocardiography was not associated with lower risk of post-operative MACE, even in a high risk population. Future guidelines should encourage pre-operative echocardiography only in specific patients with cardiovascular disease among whom findings can be translated into effective changes in care.

**Electronic supplementary material:**

The online version of this article (doi:10.1186/s12872-016-0357-5) contains supplementary material, which is available to authorized users.

## Background

Echocardiography is commonly performed prior to surgery [1], and abnormalities detected through echocardiography may be associated with higher risk of major adverse cardiac events (MACE) following surgery [2, 3]. Although the information obtained through echocardiography could inform care, in the general surgical population, pre-operative echocardiography provides little additional prognostic information, and its usefulness in guiding peri-operative treatment is unclear [2, 3]. For this reason, echocardiography is not recommended for routine pre-operative evaluation among patients without cardiovascular disease (CVD) [2–5]. In previous studies, pre-operative echocardiography has been associated with higher 30-day and 1-year mortality and longer length of stay [1, 6], which may be a result of higher risk patients being more likely to receive echocardiography. Additional testing, treatments, or treatment delays or cancellations triggered by echocardiography results may also contribute to these observations.

Preexisting CVDs, particularly heart failure (HF), valvular heart disease (VHD), and recent myocardial infarction (MI), are risk factors for MACE following non-cardiac surgery [3], and the proportion of surgical patients with CVD, including HF, VHD, and recent MI, is increasing [7]. Echocardiography is well-established as a valuable tool for diagnosis and evaluation of symptoms of many CVDs, and pre-operative echocardiography may be helpful for guiding peri-operative care among patients with established CVD [2, 3, 5, 7]. We therefore hypothesized that pre-operative echocardiography is associated with a reduced risk of post-operative MACE among patients with coronary artery disease, particularly those who also have HF, VHD, or recent MI.

We examined the associations echocardiography performed in the 3 months prior to surgery (hereafter called pre-operative echocardiography) with post-operative MACE among patients with coronary artery disease undergoing elective, non-cardiac surgery in the United States Veterans Affairs (VA) healthcare system. Because concerning findings on echocardiography may cause patients and clinicians to avoid surgery, we additionally examined the correlations between echocardiography use, surgical volume, and surgery cancellations.

## Methods

### Study population

We conducted a retrospective cohort study within the VA healthcare system during fiscal years 2000-2012. The study protocol was reviewed and approved by the local VA Institutional Review Board of each co-author and conforms to the ethical guidelines of the 1975 Declaration of Helsinki. Because this study included only data routinely collected as part of clinical care, the Institutional Review Boards approved a waiver of informed consent.

The study population has been previously described [8]. This cohort was initially assembled to examine MACE following non-cardiac surgery in patients within 2 years of placement of a coronary stent [8]. Briefly, patients undergoing placement of coronary stents within the VA healthcare system during fiscal years 2000 through 2012 were identified using International Classification of Diseases 9^th^ edition (ICD9) procedure codes 36.06 for bare metal stents and 36.07 for drug eluting stents in VA administrative data. Non-cardiac surgery in the 2 years following stent placement was identified through the VA Surgical Quality Improvement Program (VASQIP) registry. For each patient with a stent, 2 patients without stents undergoing non-cardiac surgery were included, matched on fiscal year of surgery, age within 5 years, work relative value unit (a measure of surgery complexity) within 5 units, surgical specialty, pre-operative creatinine >2 mg/dl, classification of high risk surgery (suprainguinal, intrathoracic, or intraperitoneal operations), and the components that make up the Revised Cardiac Risk Index (coronary artery disease, HF, stroke, chronic kidney disease, and insulin-dependent diabetes) [9]. All of the patients had a history of coronary artery disease. For the current study, we further limited the cohort to 8,823 stent/surgery patients and 17,818 matched surgery-only patients undergoing elective surgeries. Patients could be included in the cohort more than once if they had multiple surgeries. Patients with a history of MI in the 6 months prior to surgery or a history of HF or valvular disease in the past 24 months were considered a particularly high risk population. To examine the association between echocardiography and surgical volume and number of surgery cancellations, we examined a cohort of 126,773 individuals who had coronary stents placed, identified as described above.

### Pre-operative echocardiography

Echocardiography within the 3 months prior to surgery was identified by Current Procedure Terminology (CPT) codes (93312, 93315, 93318, 93350, 93351, 93303, 93304, 93306, 93307, 93308, 93320, 93321, 93325) and ICD9 procedure codes (88.72) in administrative data. Because a recent echocardiogram may influence the decision to perform another echocardiogram, we also identified echocardiography in the 3 to 12 months prior to surgery.

### Covariates

Covariates of interest were obtained from VASQIP (demographics, social factors, pre-operative comorbidities, and operative characteristics). Additional comorbidities were identified from the administrative data based on ICD9 diagnosis codes. For patients with surgeries in fiscal year 2005 and later, prescriptions for beta-blockers, statins, aspirin, clopidogrel, warfarin, renin-angiotensin system inhibitors, calcium channel blockers, diuretics, amiodarone, digoxin, and nitrates extending into or through the 30 days prior to surgery were identified using VA pharmacy data.

### Post-operative MACE

MACE within 30 days following non-cardiac surgery was defined as an MI recorded in VASQIP or administrative data, revascularization recorded in administrative data, or death recorded in VASQIP or administrative data. This definition of MACE has been used previously in this population [8].

### Statistical analysis

Patient and operative characteristics were compared by risk group and receipt of pre-operative echocardiography. Tests for differences conducted using Chi-square tests for proportions and Wilcoxon Rank Sum for continuous variables. These analyses were performed using SAS version 9.3.

For hospitals with at least 25 included surgeries, hospital-level echocardiography rates were calculated as the percentage of patients who received an echocardiogram in the 3 months prior to surgery. Of the 146 VA hospitals, we included 117 with at least 25 surgeries in the overall population, 87 with at least 25 surgeries in the population with recent MI, HF, or VHD, and 117 with at least 25 surgeries in the population without recent MI, HF, or VHD. To adjust the hospital-level echocardiography rates for case-mix, we constructed mixed-effects logistic regression models with the outcome of pre-operative echocardiography, random intercepts for the hospitals, and patient and operative characteristics as the independent variables [10]. Values of the random intercepts were calculated using empirical Bayesian estimates [11], and case-mix adjusted echocardiography rates for each hospital were calculated using the intercept for that hospital and the mean values of the patient and operative characteristics across hospitals. Observed and case-mix adjusted hospital-level echocardiography rates were plotted using a density plot. These analyses were repeated stratified by MACE risk group. Plots were developed using the R-program statistical software and GGPLOT2 package [12].

Linear regression was used to model MACE risk differences associated with a 10 % higher hospital-level rate of pre-operative echocardiography and by quartiles of hospital-level rate of pre-operative echocardiography. Models were repeated for case-mix adjusted rate of pre-operative echocardiography. The association between patient-level receipt of pre-operative echocardiography and MACE following surgery was estimated using logistic regression with a random intercept for hospital. Backwards stepwise selection was used to identify covariates from the patient and operative characteristics expected to be associated with echocardiography and/or MACE. All models were constructed using R-program statistical software and the lme4 package [13, 14].

In the cohort of patients who received a coronary stent, we calculated the hospital-level correlation between rate of pre-operative echocardiography use and surgical volume and number of surgery cancellations in the 2 years following coronary stent placement. Additionally, we examined the percentage of planned surgeries which were cancelled in individuals with echocardiography in the 3 months prior to the planned surgery and the percentage in the overall population.

## Results

Among 26,641 elective, non-cardiac surgeries, 4,378 (16.4 %) had pre-operative echocardiography (Table 1). Among patients with high-risk conditions (recent MI, HF, or VHD), 28.7 % had pre-operative echocardiography, compared to 12.8 % of patients without those conditions. The median time between echocardiography and surgery was 35 days (interquartile range 10-63 days). Patients who received pre-operative echocardiography were more likely to be ≥60 years of age and to have a history of diabetes, peripheral vascular disease, coronary artery bypass graft, chronic kidney disease, arrhythmia, and pacemakers. Patients with coronary stents were slightly less likely to have pre-operative echocardiography than patients without stents. Compared to patients without pre-operative echocardiography, the patients with pre-operative echocardiography were more likely to have inpatient surgery, higher work relative value units, high risk surgeries, and, among the group with coronary stents, a shorter time from stenting to surgery.

**Table 1:** Characteristics of surgical patients in the Veterans Affairs health system by whether or not they receive pre-operative echocardiography^a^

	Patients with heart failure, valvular heart disease, recent myocardial infarction				Patients without heart failure, valvular heart disease, recent myocardial infarction				
Echocardiogram in the 3 months prior to surgery	Yes (*N* = 1,732)		No (*N* = 4,307)		Yes (*N* = 2,646)		No (*N* = 17,956)		*P*-Value
Demographics									
Age									
Years, Median (25th percentile-75th percentile)	67.0	(61.0–75.0)	67.0	(60.0–75.0)	65.0	(60.0–73.0)	64.0	(59.0–72.0)	<0.001
<60	339	(19.6)	968	(22.5)	601	(22.7)	4,949	(27.6)	<0.001
≥60	1,393	(80.4)	3,339	(77.5)	2,045	(77.3)	13,007	(72.4)	
Race									
Black	194	(11.5)	451	(10.8)	315	(12.3)	1,910	(11.0)	0.11
Other	25	(1.5)	54	(1.3)	32	(1.3)	177	(1.0)	
White	1,471	(87.0)	3,686	(88.0)	2,207	(86.4)	15,321	(88.0)	
Gender									
Female	27	(1.6)	94	(2.2)	40	(1.5)	401	(2.2)	0.04
Male	1,705	(98.4)	4,213	(97.8)	2,606	(98.5)	17,555	(97.8)	
Health status									
Echocardiogram in the 3 to 12 Months Prior to Surgery									
No	1,105	(63.8)	2,318	(53.8)	2,254	(85.2)	14,561	(81.1)	<0.001
Yes	627	(36.2)	1,989	(46.2)	392	(14.8)	3,395	(18.9)	
Myocardial infarction within 6 months									
No	997	(57.6)	2,465	(57.2)	2,646	(100.0)	17,956	(100.0)	<0.001
Yes	735	(42.4)	1,842	(42.8)	0	(0.0)	0	(0.0)	
Heart failure hospitalization within 6 months									
No	1,108	(64.0)	3,115	(72.3)	2,646	(100.0)	17,956	(100.0)	<0.001
Yes	624	(36.0)	1,192	(27.7)	0	(0.0)	0	(0.0)	
History of heart failure									
No	784	(45.3)	2,381	(55.3)	1,974	(74.6)	15,444	(86.2)	<0.001
Yes	948	(54.7)	1,924	(44.7)	672	(25.4)	2,472	(13.8)	
Valvular heart disease									
No	882	(50.9)	2,329	(54.1)	2,646	(100.0)	17,956	(100.0)	<0.001
Yes	850	(49.1)	1,978	(45.9)	0	(0.0)	0	(0.0)	
Coronary artery stents									
None	918	(53.0)	2,231	(51.8)	1,905	(72.0)	12,764	(71.1)	<0.001
Bare metal stents	433	(25.0)	1,222	(28.4)	382	(14.4)	2,563	(14.3)	
Drug-eluting stents	371	(21.4)	830	(19.3)	350	(13.2)	2,563	(14.3)	
Both bare metal and drug-eluting stents	10	(0.6)	24	(0.6)	9	(0.3)	66	(0.4)	
Diabetes									
None	1,031	(59.5)	2,830	(65.7)	1,723	(65.1)	12,360	(68.8)	<0.001
Non-insulin dependent	308	(17.8)	754	(17.5)	483	(18.3)	3,165	(17.6)	
Insulin dependent	393	(22.7)	723	(16.8)	440	(16.6)	2,431	(13.5)	
Peripheral vascular disease									
No	1,063	(61.4)	2,854	(66.3)	1,760	(66.5)	13,772	(76.7)	<0.001
Yes	669	(38.6)	1,453	(33.7)	886	(33.5)	4,184	(23.3)	
Stroke									
No	1,681	(97.1)	4,230	(98.3)	2,544	(96.2)	17,673	(98.6)	<0.001
Yes	51	(2.9)	75	(1.7)	102	(3.9)	243	(1.4)	
Hypertension									
No	117	(9.2)	277	(10.1)	196	(9.9)	1,772	(14.2)	<0.001
Yes	1,154	(90.8)	2,458	(89.9)	1,784	(90.1)	10,749	(85.9)	
Coronary artery bypass grafting									
No	1,600	(92.4)	4,024	(93.4)	2,554	(96.5)	17,467	(97.3)	<0.001
Yes	132	(7.6)	283	(6.6)	92	(3.5)	489	(2.7)	
Chronic kidney disease									
No	1,268	(73.2)	3,514	(81.6)	2,229	(84.2)	16,083	(89.6)	<0.001
Yes	464	(26.8)	793	(18.4)	417	(15.8)	1,873	(10.4)	
Arrhythmia									
No	789	(45.6)	2,207	(51.2)	1,681	(63.5)	12,899	(71.8)	<0.001
Yes	943	(54.5)	2,100	(48.8)	965	(36.5)	5,057	(28.2)	
Pacemaker									
No	1,686	(97.3)	4,225	(98.1)	2,618	(98.9)	17,828	(99.3)	<0.001
Yes	46	(2.7)	82	(1.9)	28	(1.1)	128	(0.7)	
Cardioverter defibrillator									
No	1,697	(98.0)	4,235	(98.3)	2,629	(99.4)	17,870	(99.5)	<0.001
Yes	35	(2.0)	72	(1.7)	17	(0.6)	86	(0.5)	
Operative Characteristics									
Inpatient/Outpatient, *n* (%)									
Outpatient	456	(26.4)	1,536	(35.8)	751	(28.5)	8,092	(45.1)	<0.001
Inpatient	1,273	(73.6)	2,761	(64.3)	1,886	(71.5)	9,837	(54.9)	
Work Relative Value Unit									
Median (25th percentile-75th percentile)	18.2	(11.8–22.6)	15.4	(9.6–21.8)	19.6	(11.8–23.3)	15.3	(8.4–21.8)	<0.001
<10	762	(44.0)	1,887	(43.8)	1,124	(42.5)	6,821	(38.0)	<0.001
10–20	372	(21.5)	1,177	(27.3)	514	(19.4)	5,847	(32.6)	
>20	597	(34.5)	1,243	(28.9)	1,008	(38.1)	5,288	(29.5)	
High Risk Surgery									
No	1,286	(74.3)	3,332	(77.4)	1,909	(72.2)	14,417	(80.3)	<0.001
Yes	446	(25.8)	975	(22.6)	737	(27.9)	3,539	(19.7)	
Procedure Type									
Digestive	402	(23.2)	1,141	(26.5)	593	(22.4)	4,959	(27.6)	<0.001
Eye/Ear/Skin	31	(1.8)	85	(2.0)	38	(1.4)	299	(1.7)	
Genital/Urinary	206	(11.9)	607	(14.1)	285	(10.8)	2,616	(14.6)	
Musculoskeletal	272	(15.7)	828	(19.2)	475	(18.0)	4,465	(24.9)	
Nervous	34	(2.0)	119	(2.8)	88	(3.3)	709	(4.0)	
Other	50	(2.9)	102	(2.4)	78	(3.0)	422	(2.4)	
Respiratory	82	(4.7)	146	(3.4)	156	(5.9)	509	(2.8)	
Vascular	655	(37.8)	1,279	(29.7)	933	(35.3)	3,977	(22.2)	
Time to Surgery Since Stent									
Days, Median (25th percentile-75th percentile)	132.0	(63.0–370.0)	184.0	(104.5–433.0)	347.0	(92.0–515.0)	406.0	(251.0–552.0)	
None	100	(5.8)	130	(3.0)	79	(3.0)	173	(1.0)	<0.001
<6 Weeks	214	(12.4)	287	(6.7)	103	(3.9)	266	(1.5)	<0.001
6 Weeks to 3 Months	166	(9.6)	611	(14.2)	41	(1.6)	341	(1.9)	
3 to 6 Months	123	(7.1)	389	(9.0)	169	(6.4)	1,405	(7.8)	
6 to 12 Months	211	(12.2)	659	(15.3)	349	(13.2)	3,007	(16.8)	
>12 Months	918	(53.0)	2,231	(51.8)	1,905	(72.0)	12,764	(71.1)	

^a^Numbers in the table are median (25^th^ percentile-75^th^ percentile) or N (column %)

**Fig. 1:**
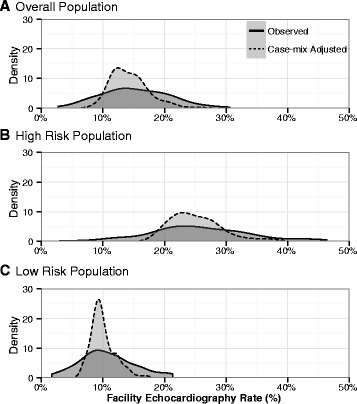
Facility variation in use of pre-operative echocardiography in the Veterans Affairs Healthcare System. Smoothed distribution of pre-operative echocardiography rates by hospital. Of the 146 Veterans Affairs hospitals, 117 with at least 25 surgeries in the overall population, 87 with at least 25 surgeries in the population with recent MI, HF, or VHD, and 117 with at least 25 surgeries in the population without recent MI, HF, or VHD were included

Before adjusting for case-mix, VA hospitals varied widely in the use of pre-operative echocardiography (mean 13.8 %, range 0.0 %–30.6 %, standard deviation 6.8 %) (Fig. 1). After adjusting for case-mix, the variability decreased (mean 14.3 %, range 8.6–25.5 %, standard deviation 3.1 %). Hospitals were more variable in the use of pre-operative echocardiography among patients with recent MI, HF, or VHD than among patients without these conditions. There was a moderate correlation (*r* = 0.53) between hospital-level use of pre-operative echocardiography among patients with and without recent MI, HF, or VHD (Additional file 1: Figure S1).

**Fig. 2:**
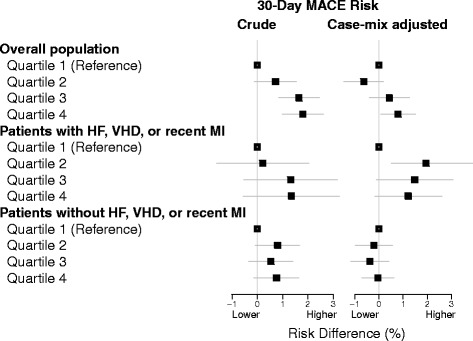
Association between hospital-level variation in pre-operative echocardiography and post-operative major adverse cardiac events. MACE: Major adverse cardiac event, HF: Heart failure, VHD: Valvular heart disease, MI: Myocardial infarction. Quartile ranges: Overall population unadjusted ≤9.72 %, 10.00–14.06 %, 14.19–18.84 %, and ≥19.01 %; Overall population case mix-adjusted ≤12.70 %, 12.75–14.20 %, 14.25–15.97 %, and ≥16.00 %; Patients with heart failure, valvular heart disease, recent myocardial infarction unadjusted ≤18.18 %, 18.46–24.86 %, 25.00–30.43 %, and ≥31.11 %; Patients with heart failure, valvular heart disease, recent myocardial infarction case mix-adjusted ≤23.45 %, 23.47–25.11 %, 25.11–27.23 %, and ≥27.49 %; Patients without heart failure, valvular heart disease, recent myocardial infarction unadjusted ≤6.06 %, 6.11–9.85 %, 9.88–12.94 %, and ≥12.95 %; Patients without heart failure, valvular heart disease, recent myocardial infarction case mix-adjusted ≤9.04 %, 9.04–9.67 %, 9.67–10.52 %, and ≥10.53 %

In the 30 days following non-cardiac surgery, 944 patients (3.5 %) experienced MACE (7.5 % with pre-operative echocardiography versus 2.8 % without pre-operative echocardiography, p < 0.001). Before adjusting for case-mix, higher hospital rates of pre-operative echocardiography were associated with higher hospital rates of MACE within 30 days of surgery (Fig. 2). For every 10 % higher rate of pre-operative echocardiography, the hospital-level 30-day risk of MACE was 1.25 % (95 % confidence interval [CI] 0.77 %, 1.74 %) higher. Associations between echocardiography and MACE were stronger comparing patients with HF, VHD, or recent MI to patients without these conditions. After adjusting for case-mix, associations were attenuated and without a clear pattern of risk across quartiles. For every 10 % higher rate of pre-operative echocardiography, the hospital-level 30-day risk of MACE was 1.02 % (95 % CI 0.07 %, 1.97 %) higher in the overall population and 1.73 % (95 % CI 0.22 %, 3.24 %) higher in the high risk population.

**Fig. 3:**
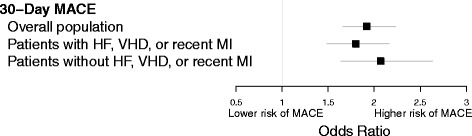
Association between patient level pre-operative echocardiography and post-operative major adverse cardiac events. MACE: Major adverse cardiac event, HF: Heart failure, VHD: Valvular heart disease, MI: Myocardial infarction. Models included a random intercept for hospital and adjusted for echocardiogram during the 3 to 12 months prior to surgery, age at surgery, recent myocardial infarction, history of heart failure, history of chronic kidney disease, history of arrhythmia, surgery case status, surgery admission status, work relative value unit, year of surgery, procedure type, and an indicator for whether the surgery was a high risk procedure

In patient-level analyses, pre-operative echocardiography was associated with a higher risk of MACE at 30 days (adjusted odds ratio 1.92, 95 % CI 1.66, 2.23) in the overall population after adjusting for risk factors for MACE (Fig. 3). The higher risk of MACE associated with pre-operative echocardiography was observed in those with and without recent MI, HF, or VHD. In sensitivity analyses, results were consistent after adjusting for pre-operative medication use among patients who had surgeries in 2005–2012 (not shown).

Hospital-level use of echocardiography was not associated with hospital surgical volume (*r* = -0.05, *p* = 0.59) or hospital number of surgery cancellations (*r* = -0.03, *p* = 0.74) in patients with coronary stents. The proportion of surgeries cancelled was 25 % in patients who received pre-operative echocardiography compared to an average cancellation rate of 21 %. The majority of cancellations (90.6 %) occurred within 7 days of the planned surgery date with 51.7 % occurring on the same day and 80.6 % occurring within 48 h.

## Discussion

In this population of veterans with coronary artery disease, 16 % underwent echocardiography in the 3 months prior to elective, non-cardiac surgery (29 % among patients with recent MI, VHD, or HF). Contrary to our hypothesis, pre-operative echocardiography was not associated with lower risk of post-operative MACE either in the overall population or in those with recent MI, HF, or VHD. In fact, both hospital-level and patient-level use of pre-operative echocardiography was associated with a higher risk of MACE.

Our results are consistent with 2 previous studies in general surgical populations. In the United States Medicare population, patients who received tests of ventricular function, including echocardiography, had higher 30-day and 1-year mortality following vascular surgery than patients who did not [6]. In a population-based study from Ontario, pre-operative echocardiography was associated with increased risk of 30-day and 1-year mortality and longer length of stay, even among patients with HF [1]. It is possible that patients would have had even higher risk of MACE without changes to management initiated following echocardiography or that the higher risk associated with echocardiography reflects patient risk factors that were not fully accounted for in statistical analysis. However, it is also possible that echocardiography led to changes or delays in care that could have contributed to the increased risk of MACE. In a prior study, patients who received pre-operative echocardiography were more likely to receive new prescriptions for beta-blockers, statins, and renin-angiotensin system inhibitors [1]. Although these medications can be beneficial for chronic management and may globally reduce cardiovascular risk, recent initiation may be harmful in some patients undergoing surgery [15].

Echocardiography is widely indicated for patients with suspected new or worsening CVD [2]. However, clinical guidelines have recommended against routine pre-operative echocardiography in patients without CVD since at least 2002 [16]. In recent years, avoiding routine pre-operative echocardiography among patients without CVD has received particular attention as part of appropriate use criteria and the Choosing Wisely campaign because of increased costs and lack of value in guiding surgical care [2–4, 17]. The guidelines and recommendations are less clear on the value of pre-operative echocardiography in patients with CVD, like the patients with coronary artery disease included in the current study. Additionally, guidelines do not always have a strong impact on patient care. A recent study of United States nationally representative data from 1997–2010 found that there was little change in the routine utilization of pre-operative radiography, hematocrit, urinalysis, and cardiac stress testing following recommendations against their use, but pre-operative electrocardiogram use did decline after professional societies recommended against it [18].

This study had several strengths, include the large population treated in an integrated health system. However, there are also important limitations to this observational, non-randomized study that prevent us from drawing conclusions about the causal effect of pre-operative echocardiography. Although we adjusted for MACE risk factors through case-mix adjustment in hospital-level analysis and multivariable models in patient-level models, there may be residual confounding by indication, where patients who receive the test have higher underlying risk. We did not find associations between use of pre-operative echocardiography and cancellations of surgeries. Nor did we find an association between hospital pre-operative echocardiography use and surgical volume. However, we could only detect cancellations of schedule surgeries; echocardiography could have resulted in surgeries never being scheduled. We could not determine whether echocardiography was performed as part of a pre-operative evaluation or for another clinical indication. In addition, results of the echocardiograms were not available. Because the data came from the VA health system, there were few women included.

## Conclusions

In conclusion, pre-operative echocardiography was common among this population of patients with coronary artery disease undergoing elective, non-cardiac surgery. Pre-operative echocardiography was not associated with a lower risk of post-operative MACE either in the overall population or in the high risk subgroup with recent MI, VHD, or HF. These results suggest that routine pre-operative echocardiography may not reduce MACE even in high risk populations. Future guidelines should encourage pre-operative echocardiography only in specific patients among whom findings can be translated into effective changes in care.

## Additional file

Additional file 1: Figure S1. Hospital-level variation in preoperative echocardiography by risk of post-operative major adverse cardiac events. (PDF 439 kb)

## Abbreviations

CI: Confidence interval
CPT: Current procedure terminology
CVD: Cardiovascular disease
HF: Heart failure
ICD9: International classification of diseases 9^th^ edition
MI: Myocardial infarction
VA: Veterans affairs
VASQIP: Veterans Affairs Surgical Quality Improvement Program
VHD: Valvular heart disease
