# Supplementary material for: Pre-operative echocardiography among patients with coronary artery disease in the United States Veterans Affairs healthcare system: A retrospective cohort study
Source: BMC Cardiovasc Disord. 2016 Sep 5;16(1):173. doi: 10.1186/s12872-016-0357-5 (PMC5011899; doi:10.1186/s12872-016-0357-5)
Supplement: Additional file 1: Figure S1. — Hospital-level variation in preoperative echocardiography by risk of post-operative major adverse cardiac events. (PDF 439 kb) [file 12872_2016_357_MOESM1_ESM.pdf]

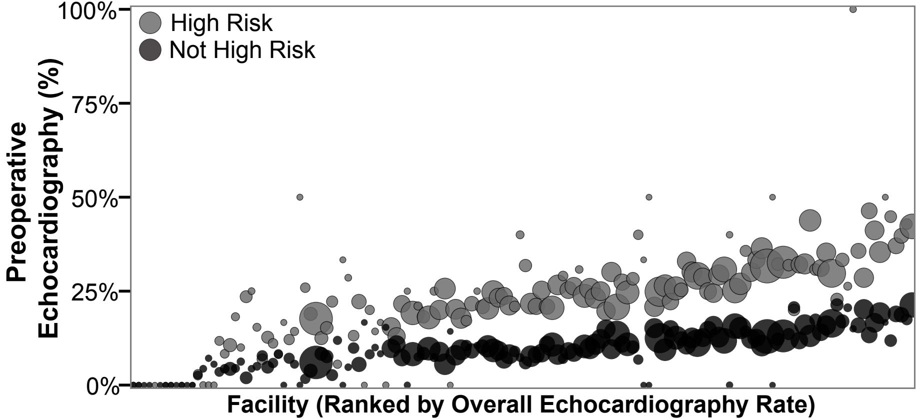

Bubble size indicates facility volume (Range 1-603)
High risk defined as recent myocardial infarction, heart failure, or valvular disease.
